# VCF‐Server: A web‐based visualization tool for high‐throughput variant data mining and management

**DOI:** 10.1002/mgg3.641

**Published:** 2019-05-24

**Authors:** Jianping Jiang, Jianlei Gu, Tingting Zhao, Hui Lu

**Affiliations:** ^1^ Department of Bioinformatics and Biostatistics, SJTU‐Yale Joint Center for Biostatistics, College of Life Science and Biotechnology Shanghai Jiao Tong University Shanghai China; ^2^ Center for Biomedical Informatics Shanghai Children’s Hospital Shanghai China

**Keywords:** NGS, software, variant filtering, variant management, VCF visualization

## Abstract

**Background:**

Next‐generation sequencing (NGS) has been widely used in both clinics and research. It has become the most powerful tool for diagnosing genetic disorders and investigating disease etiology through the discovery of genetic variants. Variants identified by NGS are stored in variant call format (VCF) files. However, querying and filtering VCF files are extremely difficult for researchers without programming skills. Furthermore, as the mutation data are increasing exponentially, there is an urgent need to develop tools to manage these variant data in a centralized way.

**Methods:**

The VCF‐Server was developed as a web‐based visualization tool to support the interactive analysis of genetic variant data. It allows researchers and medical geneticists to manage, annotate, filter, query, and export variants in a fast and effective way.

**Results:**

In this study, we developed the VCF‐Server, a powerful and easily accessible tool for researchers and medical geneticists to perform variant analysis. Users can query VCFs, annotate, and filter variants without knowing programming code. Once the VCF file is uploaded, VCF‐Server allows users to annotate the VCF with commonly used databases or user‐defined variant annotations (including variant blacklist and whitelist). Variant information in the VCF is shown visually via the interactive graphical interface. Users can filter the variants with flexible filtering rules, and the prioritized variants can be exported locally for further analysis. As VCF‐Server adopts a web file system, files in the VCF‐Server can be stored and managed in a centralized way. Moreover, VCF‐Server allows direct web‐based analysis (accessible through either desktop computers or mobile devices) as well as local deployment.

**Conclusions:**

With an easy‐to‐use graphical interface, VCF‐Server allows researchers with little bioinformatics background to explore and mine mutation data, which may broaden the application of NGS technology in clinics and research. The tool is freely available for use at https://www.diseasegps.org/VCF-Server?lan = eng.

## 1 BACKGROUND

Next‐generation sequencing (NGS) has facilitated the discovery of disease‐linked genetic variants and has been widely used for disease etiology investigation and clinical genetic diagnostics (Gong, Jiang, Duan, & Lu, 2018; Zhao & Wei, 2018). Mutations identified by NGS are usually stored in variant call format (VCF) files (Danecek et al., 2011), which have become the community standard for storing mutations data. Although VCF has been widely used in many genomics projects, such as 1,000 Genomes (Genomes Project et al., 2015), the exome aggregation consortium (ExAC) (Lek et al., 2016), and the cancer genome atlas (TCGA) (Cancer Genome Atlas Research, 2008), analysis of VCF files requires bioinformatics expertise. Querying and filtering VCFs are extremely difficult for researchers without programming skills. Medical geneticists from centers lacking bioinformatics expertise struggle to find easy‐to‐use tools for variant analysis. Furthermore, as the amount of mutation data generated from NGS is increasing exponentially, there is an urgent need to develop tools to manage these variant data.

Recently, many efforts have been made toward developing graphical tools to process VCF files for researchers with limited bioinformatics background. Tools like SNVerGUI (W. Wang, Hu, Hou, Hu, & Wei, 2012), database.bio (Ou et al., 2015), DaMold (Pandey, Pabinger, Kriegner, & Weinhausel, 2017), mirVAFC (Li et al., 2017), GAVIN (van der Velde et al., 2017), and gNOME (Lee et al., 2014) have been developed to help nonbioinformaticians prioritize variants. However, they share the disadvantage of depending on a well‐defined set of annotations, discarding user‐defined annotations in VCFs and thus limiting the user to predefined features. Experienced researchers might prefer to keep their in‐house annotations for downstream analysis. Other tools including VCF‐Miner (Hart et al., 2016), VCF.Filter (Muller et al., 2017), myVCF (Pietrelli & Valenti, 2017), and BrowseVCF (Salatino & Ramraj, 2017) skip the annotation step and allow users to filter variants with their in‐house annotations in the VCF. However, in most cases, they require users to annotate the VCF with other annotation tools before querying and filtering variants. Furthermore, they require installation and cannot manage mutation data in a centralized way.

To fill this gap, we developed the VCF‐Server, a fast and fully interactive graphical tool for variant analysis. It allows nonbioinformatician researchers to query VCFs and annotate and filter variants without the need for a single line of programming code. VCF‐Server allows users to annotate VCFs with commonly used databases or user‐defined variant annotations (including variant blacklist and whitelist). Variant information in the VCF is shown visually via the interactive graphical interface. Users can filter the variants with flexible filtering rules, and the prioritized variants can be exported to a local computer for further analysis. VCF‐Server adopts a web file system to centralize and manage VCF files, which will improve the efficiency of variant analysis. VCF‐Server allows both direct web‐based analysis (accessible through either desktop computers or mobile devices), as well as local deployment. It allows researchers with little bioinformatics background to explore and interpret mutation data, thereby fostering translational research in the field of genetics.

## 2 IMPLEMENTATION

### 2.1 Technical overview

**Figure 1:**
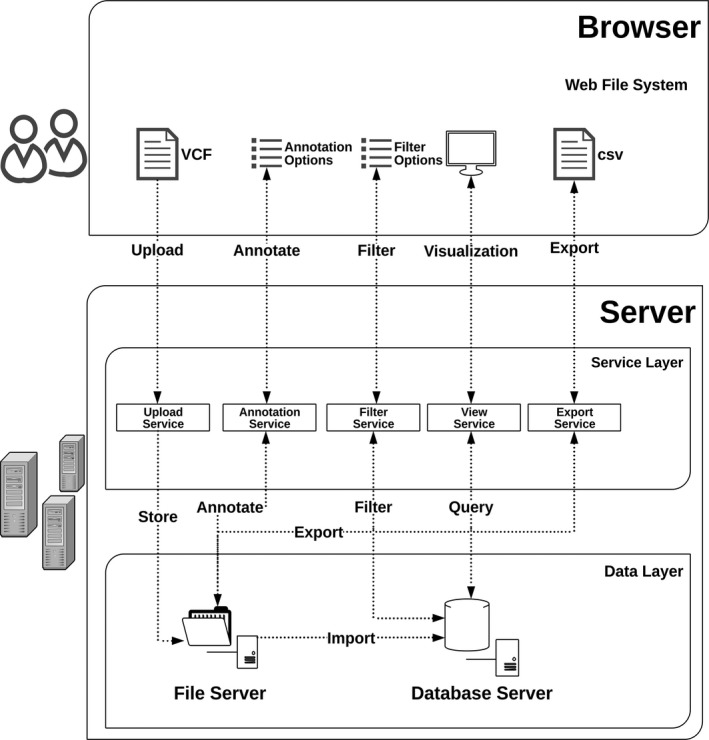
Hierarchical system design of VCF‐Server. VCF‐Server is a web application based on the Browser/Server architecture

VCF‐Server is a web application based on the Browser/Server architecture (Figure 1). The core functional modules on VCF‐Server are implemented in C to speed up VCF processing. The front end of VCF‐Server is written in Sails.js and built with the Node.js framework. The back end is implemented with PERL‐CGI. VCF‐Server supports multiple users and allows every user to work within his/her own private data space. Data transmitted from the back end to the front are encrypted to prevent data leakage. The platform works entirely on a simple web browser and is accessible through either desktop computers or mobile devices.

### 2.2 Parsing VCF files and storing variants

VCF is a standard format for storing genetic variation data in many genomics projects. The metadata in the header describes the dataset and relevant reference sources, as well as definitions of all the annotations used to qualify and quantify the properties of the variant calls contained in the VCF file. The first eight columns in the body store the identifiers of chromosome (CHROM), the 1‐based position of the variant (POS), the identifier of the variation in dbSNP (Sherry, Ward, & Sirotkin, 1999) (ID), the reference base or sequence (REF), alternative alleles at this position (ALT), quality score (QUAL), a flag indicating which of a given set of filters the variation has passed (FILTER), and a variant‐specific annotation (INFO) that describes the variant in key‐value pairs or flags (present/absent). Variant annotations from annotation tools/databases are stored in the INFO field, and multiple items are separated by semicolons. The ninth column is the format (FORMAT) column, which stores the format of the genotype fields provided for each sample. The sample columns follow the format column. The format and sample columns are optional. There is no limit to the number of samples or annotations that can be added to the VCF.

VCF‐Server can handle uncompressed or compressed (*.gz) VCF files. The modules of VCF parsing and processing on VCF‐Server are implemented in HTSlib. HTSlib is a standard C library for quickly accessing high‐throughput sequencing data and is also the core library of samtools (Li et al., 2009) and bcftools (Li, 2011). Therefore, VCF‐Server is fully compatible with variant data produced by most of the variant‐calling tools such as GATK (McKenna et al., 2010) and samtools. VCF‐Server transforms VCFs into JSON arrays and stores them in a MongoDB database. MongoDB is a schema‐free database with flexibility and efficiency of use. The metadata in a VCF is sparse and most of them are key‐value pairs, which is a perfect match for MongoDB. After loading data to MongoDB, VCF‐Server will index the commonly used fields, like CHROM and POS, to speed up querying and filtering. Users can index any field in VCF according to their needs.

### 2.3 Interactive graphical user interface (GUI)

VCF‐Server adopts a simplified web design. Most of the functional panels are hidden by default to enable a large space to display variants and annotations. VCF‐Server provides a filter panel to help nonbioinformaticians query and filter VCFs. The variants and annotations are displayed in a tabular form in the center of the browser. Each column can be hidden or displayed and can be sorted to enable the user to focus on interesting information. The tabular display is implemented using Data Tables.

The filter panel shows the filters that have been applied to the VCF and the number of variants that passed all previous filter(s). Each filter is applied to a single field selected from the filter list. VCF‐Server classifies filters into three groups: basic filters, information filters, and format filters which, respectively, corresponds to the first five fields (REF and ALT fields are excluded), the INFO field and the sample fields in a VCF. VCF‐Server parses metadata from VCFs and automatically generates applicable operators according to data types. Numeric (including integer and float type) fields are filtered using relational operators such as “>” and “<”. Binary (flag type) fields are filtered based on their “1” or “0” status. For string fields, the interface will automatically switch to “select box.” The adding and removing of filters follow the last‐in‐first‐out (LIFO) rule, and the new filter will be applied to the variants that have passed all previous filter(s).

Basic filters and information filters will be applied to all samples, and format filters will be applied to user‐selected samples. This feature may be useful in the analysis of multiple samples. For instance, autosomal recessive variants in a trio can be selected by requiring parents to be heterozygous and the affected child to be homozygous. The flexible sample filter combined with other filters allows users to create flexible filter rules, which may extend the application of VCF‐Server to complex biological research.

### 2.4 Centralized VCF management

Next‐generation sequencing technology has moved biology into the Big data era, and huge amounts of sequencing data are generated by sequencing centers every day. After initial bioinformatics processing, the information in sequencing data will be transformed to variants and stored in VCFs. As sequencing is widely used in daily research and clinics, researchers have received many VCF files from sequencing organizations.

The huge amount of VCFs and variant data poses a challenge to data management for genomics scientists and medical geneticists (Carson, Liu, Lu, Jia, & Lu, 2017). However, most of the VCF mining tools focus on VCF visualization and filtering, but few tools are available to address this problem. An effective VCF management system can save a tremendous amount of time for researchers in variant analysis. VCF‐Server adopts a web file system and manages VCF files in multiple projects. VCF files are clustered into different projects, which will improve the efficiency of file tracking. VCF‐Server allows users to create different projects and put related VCFs in them. The uploaded VCFs can be removed by the owner and will be cleared from VCF‐Server after deletion. VCF‐Server allows users to search VCF files within their data space.

### 2.5 Variant annotation with commonly used databases

Following variant calling, functional annotation is a crucial step for determining the pathogenicity of a variant. There are many tools available for variant annotation, such as ANNOVAR (Wang, Li, & Hakonarson, 2010), VEP (McLaren et al., 2016), SnpEff (Cingolani, Platts et al., 2012), etc. VCF‐Server can annotate the uploaded VCF files with commonly used databases (Table S1), like refGene (O'Leary et al., 2016) and ClinVar (Landrum et al., 2014). The annotation function of VCF‐Server is not meant to replace other annotation tools but to be a supplementary tool. The annotation function aims to facilitate users if the VCF does not contain a specific annotation. Therefore, different from other tools, VCF‐Server annotates VCFs with as little annotations as possible in order not to add unnecessary information to the VCF. For example, VCF‐Server allows users to annotate a VCF with only a SIFT_score (Ng & Henikoff, 2003) from dbNSFP (Liu, Wu, Li, & Boerwinkle, 2016) instead of adding all of the 36 annotations to the VCF. For example, researchers can select the allele frequency of East Asians in the 1,000 Genome Project (Genomes Project et al., 2015) to annotate Asian samples. The annotation databases on VCF‐Server are classified into four groups: variant functions, frequency databases, prediction databases, and customized annotations. VCF‐Server allows users to upload annotations in VCF format to build customized annotation databases. These customized annotation databases are private and only accessible to the uploader. Tools used on VCF‐Server for annotation are ANNOVAR (Yang & Wang, 2015), SnpSift (Cingolani, Patel et al., 2012), and vcfanno (Pedersen, Layer, & Quinlan, 2016).

### 2.6 Variant filtering with user‐defined variant whitelists and blacklists

Variant whitelists and blacklists on VCF‐Server are sets of variants that are related to specific diseases or symptoms, such as genetic variants for targeted cancer therapy or variants related to the risk of Parkinson's disease. The lists of variants can be published by authorities or be curated from literature and clinical trials by the individual researchers. Variant whitelists and blacklists have many potential applications in genomics analysis. For example, screening of lung cancer patients for targeted drugs by genetic tests and screening newborns for underlying genetic diseases by sequencing.

Variants used for whitelists and blacklists should be stored in VCF format. After uploading to VCF‐Server, the list will appear in customized annotation database. Lists are stored in VCF‐Server and can be applied to all VCFs. The uploaded lists are private and can only be accessed and managed by the owner. After annotating the list, variants can be filtered according to the flags labeled by whitelist or blacklist.

### 2.7 Exporting filtered variants

The uploaded VCF and annotated VCF are stored on the VCF‐Server online file system and can be downloaded locally. All variants that have passed the filter(s) can be exported as a comma‐separated text (CSV) file that is compatible with spreadsheet programs like Microsoft Excel. The filtered variants could also be exported to other file formats, such as VCF and text.

## 3 RESULTS

### 3.1 Overview of VCF‐Server

**Figure 2:**
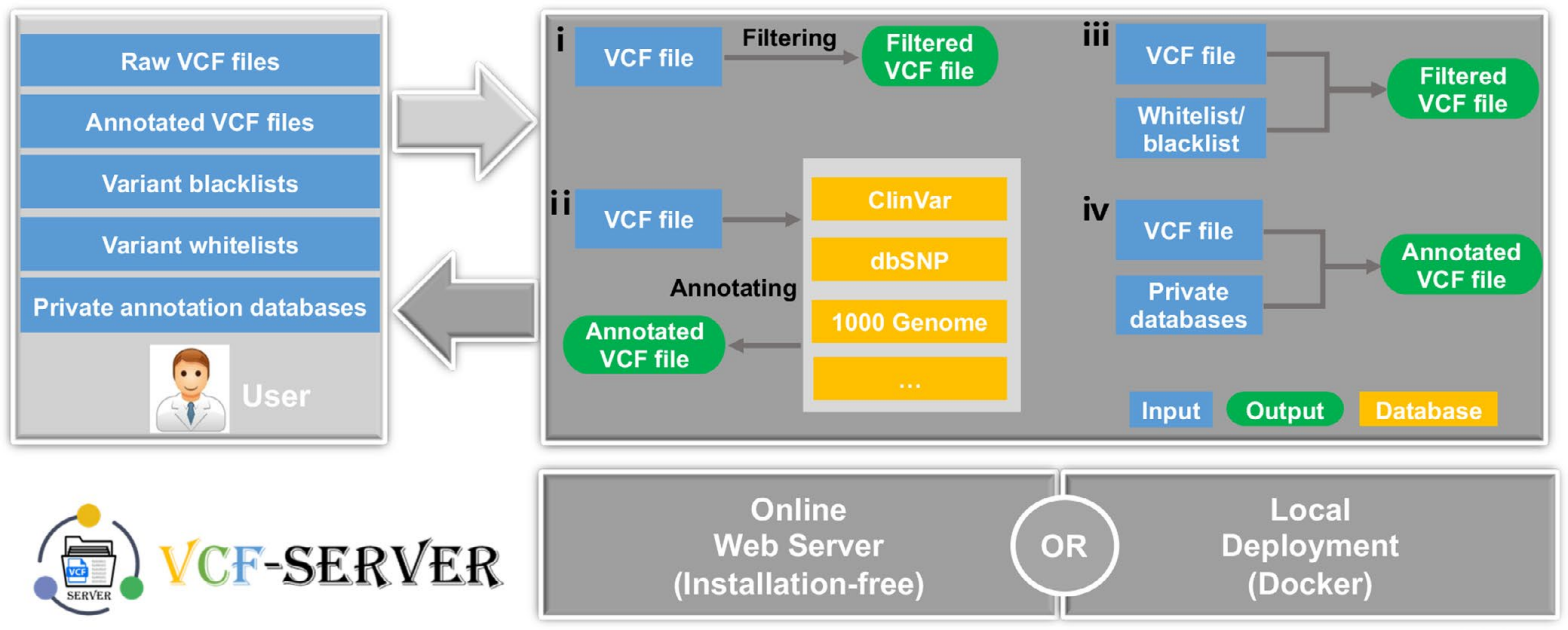
VCF‐Server workflow. A public server has been provided for easy access, and local deployment is also possible. The user uploads one or more VCF files with or without whitelists/blacklists or private annotation databases. The back end stores data separately for each individual user and invokes different modules on demand. ⅰ, ⅱ, ⅲ and ⅳ are different application scenarios on the VCF‐Server

VCF‐Server is by far the most comprehensive GUI‐based VCF mining tool that requires no programming knowledge. Without writing a single line of code, users can query VCFs, annotate, and filter variants and visualize results via the interactive graphical interface (Figure 2). It allows both direct web‐based analysis (accessible through either desktop computers or mobile devices at https://www.diseasegps.org/VCF-Server?lan = eng) as well as local deployment (Docker can be found at https://github.com/biojiang/VCF-Server-docker). Using the Docker version, VCF‐Server provides great data security, as all analyses are performed on local infrastructure without the need to upload sensitive genetic data through open networks to publicly available analysis tools. The project is fully open source, and its source code can be found at https://github.com/biojiang/VCF-Server. VCF‐Server adopts hierarchical system design and can be easily integrated into other projects. VCF‐Server is supported by all modern web browsers, including Firefox and Chrome. As the tool runs on the web server's back end and supports multiple users, it can be installed site‐wide within an organization, thereby enabling shared use.

We have systematically compared VCF‐Server with four existing VCF visualization and mining tools to demonstrate its versatile functions (Table 1). VCF.Filter is a standalone Java application that focuses on variant filtering and family analysis. The VCF visualization in VCF.Filter is not user‐friendly, and the output lacks easy readability for nonbioinformaticians. Furthermore, VCF.Filter requires the VCF to be sorted and indexed before loading to the program. myVCF implements a variant management and query application; however, variant filtering needs users to be familiar with filtering expressions. BrowserVCF and VCF‐Miner are two tools that focus on variant filtering. However, BrowserVCF does not save the data for access again at a later time, thus requiring users to reload, reimport, and reindex the same VCF each time they access the tool. VCF‐Miner requires login with an administrator account and only supports one user. Furthermore, none of these tools can manage mutation data in a centralized way and do not allow the user to annotate, query, and filter variants with user‐defined variant blacklist and whitelist. VCF‐Server overcomes these limitations and implements a number of enhanced functionalities, making it more flexible than other freely available tools (Table 1).

**Table 1:** Comparison of existing noncommercial visualization and mining tools for VCF

Features	VCF‐Server v1.0	BrowseVCF v2.8	VCF‐Miner version 2017–06−29	myVCF v1.0	VCF.Filter version 2017–04−28
VCF index required	×	×	×	×	√
Flexible variant filtering/selection	√	√	√	×	√
**Variant annotation**	√	×	×	×	×
**Variant screening**	√	×	×	×	×
**VCF management**	√	×	×	×	×
Output view customize	√	×	√	×	×
Output format	HTML, VCF, CSV	HTML, TSV	HTML, VCF, TSV	HTML	Text, VCF
Docker container	√	×	√	×	×
**Web service**	√	×	×	×	×
**Data encryption**	√	×	×	×	×
Data Persistence^a^	√	×	√	√	×
**Multiple users support**	√	×	×	×	×
Login required	×	×	√	×	×
Multiple tasks support	√	×	√	×	×
Installation required	×	√	×	√	×
Dependency for installation	×	√	×	√	×
Fully open source	√	√	×	√	√
Application architecture^b^	B/S	B/S	B/S	B/S	Stand‐alone
Graphical user interface engine	HTML + Node.js	HTML + Python‐CGI	HTML + Java	HTML + django	Java GUI
VCF parser	C/HTSlib	Python/wormtable	Java	Python/PyVCF	Java/HTSJDK
Database engine	MongoDB	Berkeley DB	MongoDB	SQLite	None
Citation		Salatino & Ramraj, 2017	Hart et al., 2016	Pietrelli & Valenti, 2017	Muller et al., 2017

Note

The highlighted features (bold) are unique for VCF‐Server.

a Data Persistence: Storing variants and VCFs on database for fast and multiple‐user access.

b B/S: Browser/Server architecture.

### 3.2 Upload data

**Figure 3:**
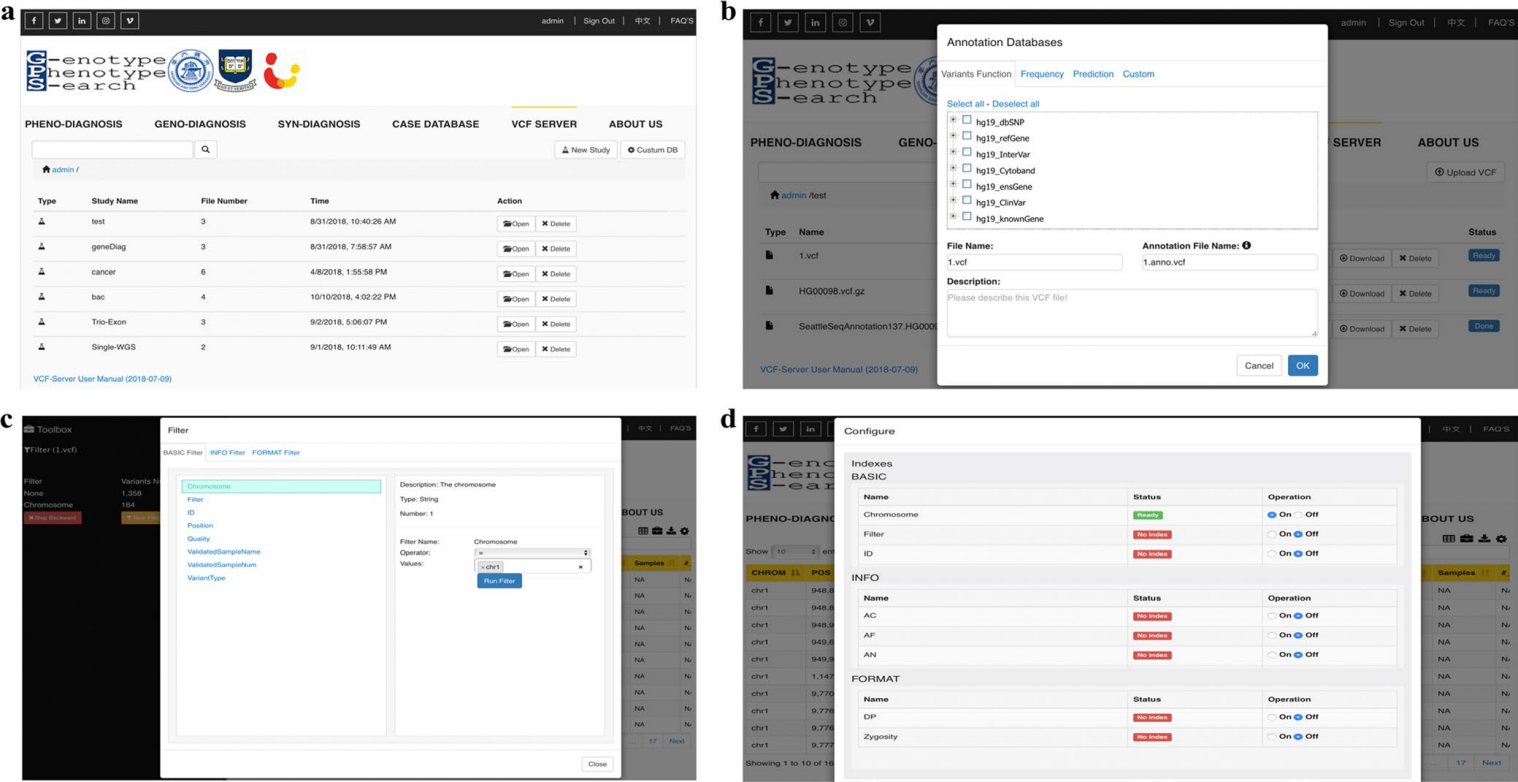
Screenshots of different functional modules on VCF‐Server. (a) VCF file management online. (b) Annotating VCF with commonly used annotation databases. (c) Variant filtering and visualization. (d) VCF index management

VCF‐Server accepts one or more VCF files as input. Each VCF file may be annotated by different annotation tools like ANNOVAR, SnpEFF, or Variant Effect Predictor (VEP). Users can upload multiple VCF files sequentially. After uploading, VCF‐Server will parse these VCF files and simultaneously import them to the database. Currently, VCF‐Server accepts either hg19/GRCh37 or hg38/GRCh38 version of the human genome for downstream functional annotation and analysis. After loading to the database, users can query, annotate, and filter these VCF files (Figure 3). The VCF files only need to be loaded once and will be stored for further analysis. All of the data and files are stored in their own data space and can be cleared by the owner.

### 3.3 Benchmark of VCF parsing, indexing, and querying

We tested the performance of VCF‐Server on three different file types: an exome trio, a whole‐genome trio, and the whole genome of sample NA12878 (v3.3.2) that was generated by the “Genome in a Bottle Consortium” (Zook et al., 2014). As shown in Table 2, on VCF‐Server, the amount of time required to process a VCF file is <20 s for standard trio exome data and the first query takes usually <5 s. The amount of time required for VCF‐Server to process a trio whole‐genome VCF is <11 min and the first query usually takes <10 s. The table also shows the amount of time taken for two similar tools, VCF‐Miner and BrowseVCF, on the same hardware. Overall, VCF‐Server is the fastest tool for processing a trio whole‐genome sequencing (WGS) or whole‐exome sequencing (WES) VCF file.

**Table 2:** Performance of VCF‐Server, BrowseVCF, and VCF‐Miner on exome and whole‐genome data

VCF Files	Size (Mb)	Variants	Step1: Pre‐processing^a^			Step2: Indexing^b^	Step3: Filtering^c^
			L0	L1	L2		
VCF‐Server							
GIAB_v.3.3.2.NA12878.vcf.gz	129	3,775,119	2m2s	3m1s	**3m14s**	**2m**	**4s**
WES.Trio.vcf.gz	5.7	129,131	4s	7s	**9s**	**5s**	**2s**
WGS.Trio.vcf.gz	236	4,852,720	3m3s	5m32s	6m30s	**2m5s**	**6s**
WGS.Trio.anno.vcf.gz	287	4,852,720	4m13s	6m43s	8m5s	**2m24s**	**6s**
BrowseVCF							
GIAB_v.3.3.2.NA12878.vcf.gz	129	3,775,119	3m24s			21m53s	3m59s
WES.Trio.vcf.gz	5.7	129,131	10s			58s	11s
WGS.Trio.vcf.gz	236	4,852,720	**4m59s**			13m49s	7m37s
WGS.Trio.anno.vcf.gz	287	4,852,720	**7m7s**			37m31s	14m10s
VCF‐Miner							
GIAB_v.3.3.2.NA12878.vcf.gz	129	3,775,119	12m2s			24m40s	10s
WES.Trio.vcf.gz	5.7	129,131	25s			27s	**2s**
WGS.Trio.vcf.gz	236	4,852,720	17m6s			28m20s	**6s**
WGS.Trio.anno.vcf.gz	287	4,852,720	20m30s			41m55s	12s

Note

All of the benchmarks are calculated on an Ubuntu Linux v16.04 server with INTEL CPU at 2.6 GHz and 64 GB RAM. All operations were performed using only one CPU. Bold value indicates which method is the fastest one of the step.

a Step required to parse and import the input VCF file into the database. L0, L1, L2 stand for different fields of the VCF file that the VCF‐server parses and imports into the database. When L0 is on, the CHROM, POS, ID, REF, ALT, QUAL, and INFO fields will be parsed and imported into the database. When L1 is on, the L0 fields and the FORMAT string will be parsed and imported into the database. When L2 is on, the VCF‐Server will parse and import all of the fields into the database.

b Indexes were built for the following fields: CHROM + POS, ID, REF + ALT, QUAL, FILTER.

c Query executed on the FILTER field, keeping only PASS variants.

### 3.4 Application example

Neonatal screening, genetic testing for targeted cancer therapy, and genetic disease diagnosis are the three common scenarios in genomic analyses, and all of them can be done on VCF‐Server. For the first two, users can first curate disease‐related variants as an annotation database and annotate the sequenced samples with the database. In the filtering step, disease‐related variants in the samples will be screened by adding a filter of “database annotated variants.” These can be extended to other genetic screenings with known variants. In the scenario of genetic disease diagnosis, a trio family with an affected child will usually be sequenced. In this situation, the inheritance pattern of disease‐causing variant(s) may be autosomal or X‐linked recessive, compound heterozygous, or a de novo germ line mutation. Assuming that the disease‐causing variants are autosomal or X‐linked recessive, VCF‐Server can return all of the putative variants in which the mother and father are heterozygous but the affected child is homozygous. Another effective way to filter variants by inheritance pattern is annotating variants with tools like MAPPIN (Gosalia, Economides, Dewey, & Balasubramanian, 2017) and then filtering them with the annotations‐of‐inheritance pattern. By adding other different filters, such as allelic frequencies from the 1,000 Genomes Project, ExAC or ESP6500 and pathogenicity predictions from SIFT or PolyPhen (Adzhubei et al., 2010), users can quickly narrow down putative variants to a small number.

To demonstrate the utility of VCF‐Server, we present an example of trio‐based genomic analysis. In this case, the genomes of the mother, father, and affected son were sequenced by WES. After joint variant calling by GATK, we detected 129,131 variants in these three genomes. There were 110,079 variants detected in the affected son's genome. Then, we annotated the VCF with refGene, ClinVar, ExAC_EAS, ESP6500_EAS, 1000Genomes_EAS, SIFT, and PolyPhen2 on the VCF‐Server. When we added the filters of “1000Genomes_EAS <0.01,” “ExAC_EAS <0.01,” and “ESP6500_EAS <0.01,” the number of variants decreased to 28,408. Restricting variants to those in exonic regions that were nonsense or missense mutations, this number dropped to 4,525. Assuming the inheritance mode of disease‐causing variants is recessive, we ultimately reduced the number of variants that passed our filters to 120. The whole analyses, including loading, annotating, and filtering, were accomplished on VCF‐Server within 30 min. Users can annotate VCF with other databases on VCF‐Server or other publicly available annotation tools and apply different filters in practical analyses.

## 4 CONCLUSION

VCF‐Server is by far the most comprehensive GUI‐based VCF visualization and mining tool with no programming‐knowledge requirement. It allows users to manage, annotate, filter, query, and export variants in a fast and effective way. The application can process any VCF file as long as it is properly formatted. VCF‐Server does not constrain the user to use a specific set of annotations and offers many interactive features to allow routine computational procedures with a great amount of flexibility. VCF‐Server adopts a web file system to store and manage VCF files in a centralized way. Furthermore, VCF‐Server supports multiple users, and each user works within his/her own data space. Owing to its user‐friendly interface, VCF‐Server is a tool that can be used by both bioinformaticians and people with no computer programming experience. We expect that this tool will empower genomics researchers and medical geneticists by reducing their dependence on bioinformatics expertise, allowing them to focus more on explaining the genomic variants that they identify.

## AVAILABILITY OF DATA AND MATERIALS

Project Name: VCF‐Server. Homepage: https://www.diseasegps.org/VCF-Server?lan=eng. Docker: https://github.com/biojiang/VCF-Server-docker. Source code: https://github.com/biojiang/VCF-Server. OS: Linux, OS X, Windows. Programming language: C, PERL‐CGI, JavaScript. License: GPL‐3.0.

## CONFLICT OF INTERESTS

The authors declare that they have no competing interests. The VCF‐Server is freely available, but the copyrights of all the annotation databases on VCF‐Server belong to the original copyright owners or organizations. If used for commercial purpose, the user must acquire the license(s) of annotation database(s) for commercial entities.
